# COVID salivary diagnostics: A comparative technical study

**DOI:** 10.1002/jmv.27883

**Published:** 2022-06-02

**Authors:** Hanh Nguyen‐Kim, Christiane Beckmann, Maurice Redondo, Jérémy Ziliox, Virginie Vallet, Karin Berger‐Sturm, Jan Von Overbeck, Lavinia Alberi Auber

**Affiliations:** ^1^ Swiss Integrative Center for Human Health Fribourg Switzerland; ^2^ Viollier Baselland Switzerland; ^3^ Red Cross Switzerland Bern Switzerland; ^4^ Task Force COVID, Canton of Bern Bern Switzerland

**Keywords:** heat‐released RNA, nasopharyngeal swabs, RNA extraction, saliva, SARS‐CoV‐2

## Abstract

Since the beginning of the coronavirus disease 2019 (COVID‐19) pandemic, molecular diagnostics of severe acute respiratory syndrome coronavirus 2 (SARS‐CoV‐2) have taken center stage in the detection of infected individuals for isolation purposes but also in the mass surveillance as a preventive strategy to contain the virus spread. While nasopharyngeal swabs (NPS) have remained the golden standard substrate, salivary diagnostic for SARS‐CoV‐2 has been proposed as an alternative and noninvasive measure in vulnerable individuals. Nevertheless, there is a widespread assumption that salivary reverse‐transcription polymerase chain reaction (RT‐PCR) does not match the quality of testing using NPS and particular care should be taken in respect to food or beverage intake, when sampling saliva. Our study indicates that without any precaution in the selection of 190 patients, nor restriction over the time window of sampling, there is 99% match in the COVID‐19 positivity between NPS and saliva when using RT‐PCR, with a reported Delta in thermal cycles (Cts) values for the viral genes Envelope (E) and Open reading frame 1ab (Orf1ab) between 0 and 2, a 98.7% sensitivity and 100% specificity. This high accuracy is maintained in pooling configurations that can be used for mass‐testing purposes in professional and educational settings. The further advantage to using crude saliva as compared to NPS or mouthwash is that direct methods yield robust results. Overall, our study validates and promotes the use of salivary diagnostic for COVID‐19 eliminating the need of a medical practitioner for the sampling, resolving the unpleasantness of the NPS intervention and empowering the patient to do self‐testing in times of need.

## 1 INTRODUCTION

In December 2019, unexplained severe pneumonia cases occurred in Wuhan, China. This outbreak was confirmed to be caused by a novel coronavirus (CoV) related to severe acute respiratory syndrome coronavirus (SARS‐CoV) and Middle East respiratory syndrome (MERS‐CoV).^1^, ^2^ The new CoV leads to symptoms resembling that of the severe acute respiratory syndrome (SARS‐CoV) from 2002 to 2003 and shares the same receptor, angiotensin‐converting enzyme 2 (ACE2) to invade the host. The new virus was named SARS‐CoV‐2, while the World Health Organization (WHO) named this illness, coronavirus disease 2019 (COVID‐19). CoVs cause systemic infections in mammals with a trend of crossing species barriers, resulting in epidemics. In humans, CoVs may cause severe clinical symptoms and high mortality depending on the variant. The vast and heterogenous propagation pattern with slow but progressive transmission in many countries and exponential growth in others can be explained by superspreader events, where urban‐dwelling asymptomatic or oligosymptomatic individuals with high viral load can infect others via airway droplets.^3^ In face of the virus morbidity, its unknown pathogenesis, and the absence of targeted therapies, by March 2020 confinement and social distancing measures were adopted in 118 countries including Switzerland. Even after the introduction of vaccination nation‐wide in 2021, with looser confinement measures, many countries have adopted mass testing and contact tracing through mobile application interfaces to keep infections at bay and gain control over new hotbeds. Switzerland has successfully implemented the FOPH guidelines on the virus control and performs more than 1400 tests/million inhabitant being one of the most diligent countries in mass testing. Now 2 years into the pandemic, with the sequel of four variants, alpha, beta, delta, and omicron, surveillance using noninvasive measures is paramount to quickly identify novel virus strains.

Nasopharyngeal swabs (NPS) have been widely used as collection material in first‐line diagnostics of COVID‐19. Besides the invasiveness of the approach, limiting the repeatability of the testing, in particular, for vulnerable age segments, the NPS vary significantly based on the operator, the site of sampling, and the viral load of the swabbed tissue.^4^ The shortage of NPS as collection agents profoundly affects the sensitivity of reverse‐transcription polymerase chain reaction (RT‐PCR) testing resulting in up to 30% of false negatives. There is numerous evidence that saliva could be used as an alternative and noninvasive biofluid suitable for SARS‐Cov2 genetic diagnostics.^5^, ^6^, ^7^ Saliva is a peripheral biofluid produced by the parotid, sublingual, and submandibular glands and rich in DNA, RNA, and proteins. The salivary glands, in particular the parotid glands due to proximity of the nasopharyngeal tract, function as a sink for respiratory pathogenic species that are released in saliva making this biofluid ideal for pathogen testing. Furthermore, recent evidence indicates that the time or method of sampling does not interfere with the results of the genetic test, and saliva can be kept at 4°C for 24 h without degradation giving great flexibility in the biofluid sampling and triaging.^8^ We have also previously demonstrated that the saliva can be used as a microbial classifier for dementia progression^9^ and it is also found to detect Influenza viruses A and B.^10^ This and other reports strongly support the use of saliva for detecting infectious diseases. Several Universities and Institutions around the world have demonstrated that saliva COVID‐19 diagnostic is the only acceptable way of routine monitoring and controlling the virus spread by performing more efficient contact tracing in the long run^11^ and preparing for the flu season in the fall/winter. Based on the accumulated evidence and with the outbreak of the highly infectious Omicron variant, in the winter of 2021, the CDC has recommended the use of saliva testing over NPS, to facilitate mass‐testing.^12^

The primary objective of this study has been to evaluate whether saliva is a surrogate biofluid to NPS in symptomatic individuals without control about the collection time or oral care and to assess the clinical performance and analytical sensitivity of RT‐PCR detection of SARS‐Cov‐2 in saliva compared to that of NPS. Secondly, our study aimed to compare the simple heat‐released RNA protocol (extraction‐free) to the magnetic beads and spin column RNA extraction protocol followed by RT‐qPCR in both NPS and saliva samples. Finally, we evaluated the sensitivity of different pooling strategies for saliva samples in detection of SARS‐CoV‐2 and assessed an optimal pool size for mass testing in public establishments, according to the measure adopted by the Swiss government during the pandemic surveillance.

## 2 MATERIALS AND METHODS

### 2.1 Participants

**Figure 1:**
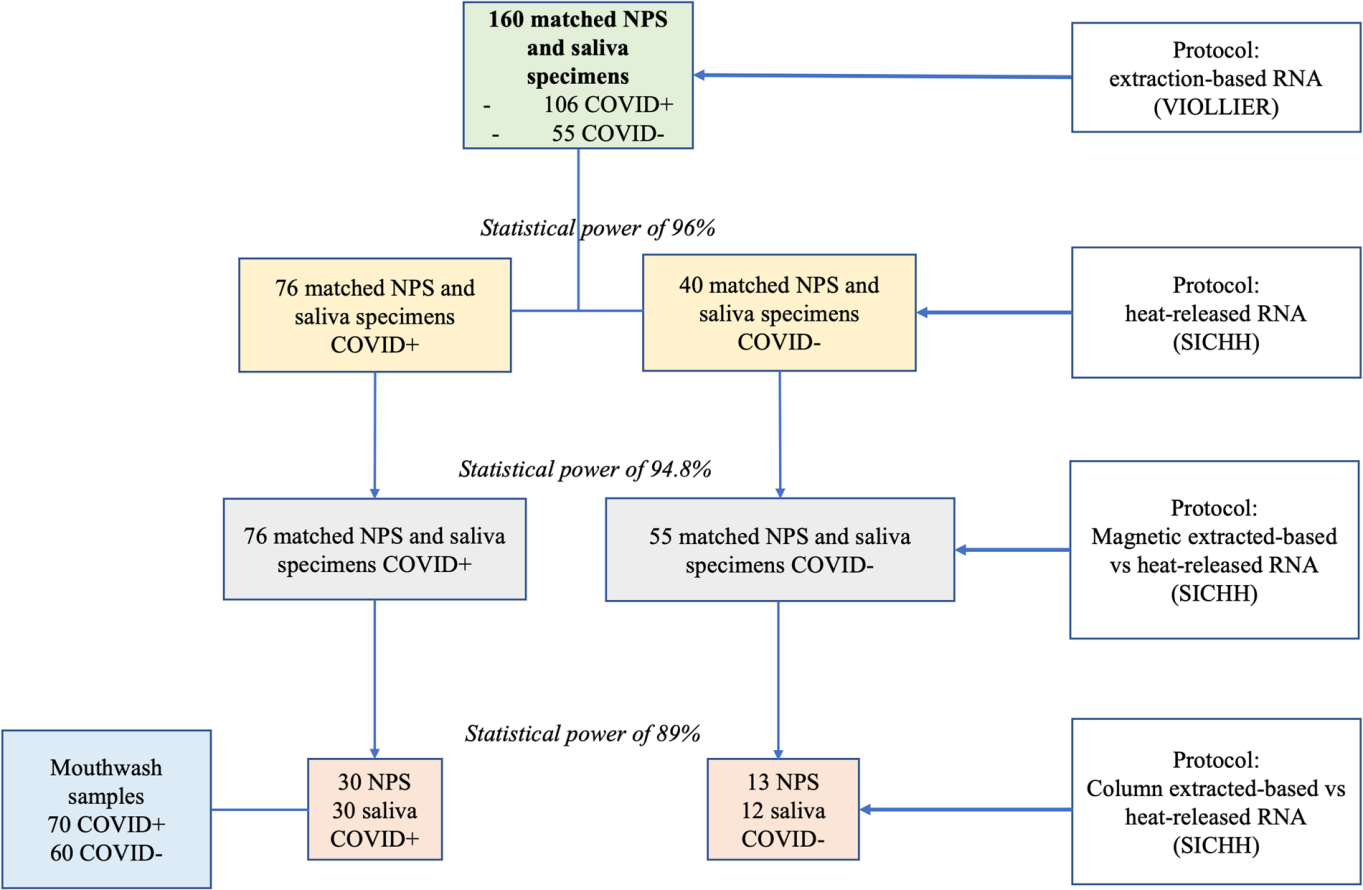
Flow chart describing the decision tree model for sample analyses. Statistical power (%) was counted based on the correlation using software G*Power 3.1. COVID, coronavirus disease; NPS, nasopharyngeal swabs

Recruitment of participants was performed at the Drive‐in Bern on a voluntary basis from January 13, 2021 to February 28, 2021. Symptomatic subjects underwent first sampling of NPS and then auto‐collected saliva (SwissEthics N. ID 2020‐02633) (Figure 1). The age segment is 18–85 years of age in both females and males. Our inclusion criteria were patients with mild to moderate acute respiratory symptoms (corona check criteria). Our exclusion criteria were patients with severe respiratory symptoms, inability to give consent, inability to follow procedures or insufficient knowledge of project language. Severity of the respiratory disease was not reported. The viral prevalence of SARS‐Cov‐2 in this period was between 2% and 10% in the tested incoming patients.

### 2.2 Sample collection

NPS were performed on all subjects following the routine procedure^13^ and sent twice to the laboratory for analysis and stored at −80°C for later analysis.

For saliva collection, subjects were asked to visualize a lemon to stimulate salivation and donate saliva. A volume corresponding to 1–2 ml of whole unstimulated saliva was collected in 50 ml sterilized conical tubes and placed in cool boxes. The collected saliva once arrived in the lab was stored at −80°C for later analysis.

### 2.3 Laboratory testing

NPS and saliva samples were processed with three different protocols for releasing RNA from samples in two different labs at VIOLLIER (Seegene extraction protocol) and SICHH (column extraction and heating protocols) (Figure 1): RNA extraction using the robot Nimbus (Seegene), RNA extraction on column (Norgen) and heat‐released RNA following an adaptation of the direct saliva method.^14^ The first two protocols follow the manufacturer instruction, while in the latter protocol, samples were diluted in Tris‐borate‐EDTA buffer (TBE) at a ratio of 1:1, followed by heat treatment at 95°C for 30 min and addition of Tween 20 to a final concentration of 0.5%.

Real‐time one‐step RT‐qPCR analysis of both extracted and heated RNA was subsequently applied using three commercial RT‐PCR kits: the AmpliCube Coronavirus SARS‐CoV‐2 (Mikrogen), the Novel Coronavirus (2019‐nCoV) Real‐Time Multiplex RT‐PCR Kit (Liferiverbiotech) and the RT‐PCR kit Allplex™SARS‐CoV‐2 Assay (Seegene) using the protocol recommended by the manufacturer either on MIC (Biomolecular System) or Lyght Cycler (Roche, Switzerland). The AmpliCube Coronavirus SARS‐CoV‐2 kit targets genomic regions of SARS‐CoV‐2: the envelope (E gene) and ORF1ab gene. The Novel Coronavirus (2019‐nCoV) detects three genes: the envelope (*E*) gene, the nucleocapsid (*N*) gene, and the ORF1ab gene. Whereas, the Allplex‐COV detects besides the envelope (*E*) gene, and the nucleocapsid (*N*) gene, the RNA‐dependent RNA polymerase (RdRp)/spike (*S*) gene and the nucleocapsid (*N*) gene. Both kits include an exogenous RNA‐based internal control (IC) to monitor the processes from nucleic acid extraction to fluorescence detection.

### 2.4 Pooling saliva samples for mass population screening using heat‐released RNA protocol

We performed the pooling dilution in 3 independent experiments using saliva specimens with Ct values from 23 to 27 for Gene E, typically observed in the population of symptomatic individuals. The dilutions were an ascending order 1:1; 1:5; 1:25, 1:64, and 1:100, respectively. The pooled samples were then processed with the heat‐released RNA protocol followed by RT‐PCR analysis by using two different kits: AmpliCube Coronavirus SARS‐CoV‐2 RT‐PCR kit from Mikrogen and Novel Coronavirus RT‐PCR Kit from Liferiver.

In another set of experiments, we tested and compared the Ct values between 60 saliva pools of 8 containing one positive specimen with that of the respective individual specimens. One positive saliva specimen was mixed with seven negative saliva specimens to create a pool of 8. RNA of the pools and each positive specimen was then collected by using heating protocol followed by RT‐PCR analysis using AmpliCube Coronavirus SARS‐CoV‐2 RT‐PCR kit from Microgen.

### 2.5 Statistical analysis

Graphical projections and statistical analysis (*p* value, R) were performed by using the software Rstudio (version 1.4.1717). A bivariate Pearson correlation was used to test where there is a statistically significant linear relationship between the Ct values of saliva and NPS samples. The Wilcoxon test was used to compare the differences in Ct values between saliva and NPS samples; pools vs individuals. *p* Values < 5% were considered statistically significant.

Percentages of analytical sensitivity, specificity, positive predictive value, and negative predictive value with their 95% confidence intervals (CI) were calculated using the “Jeffreys” method.

## 3 RESULTS

### 3.1 Comparison of SARS‐CoV‐2 detection using directly matched saliva and NPS specimens with extraction‐based RNA protocol

**Figure 2:**
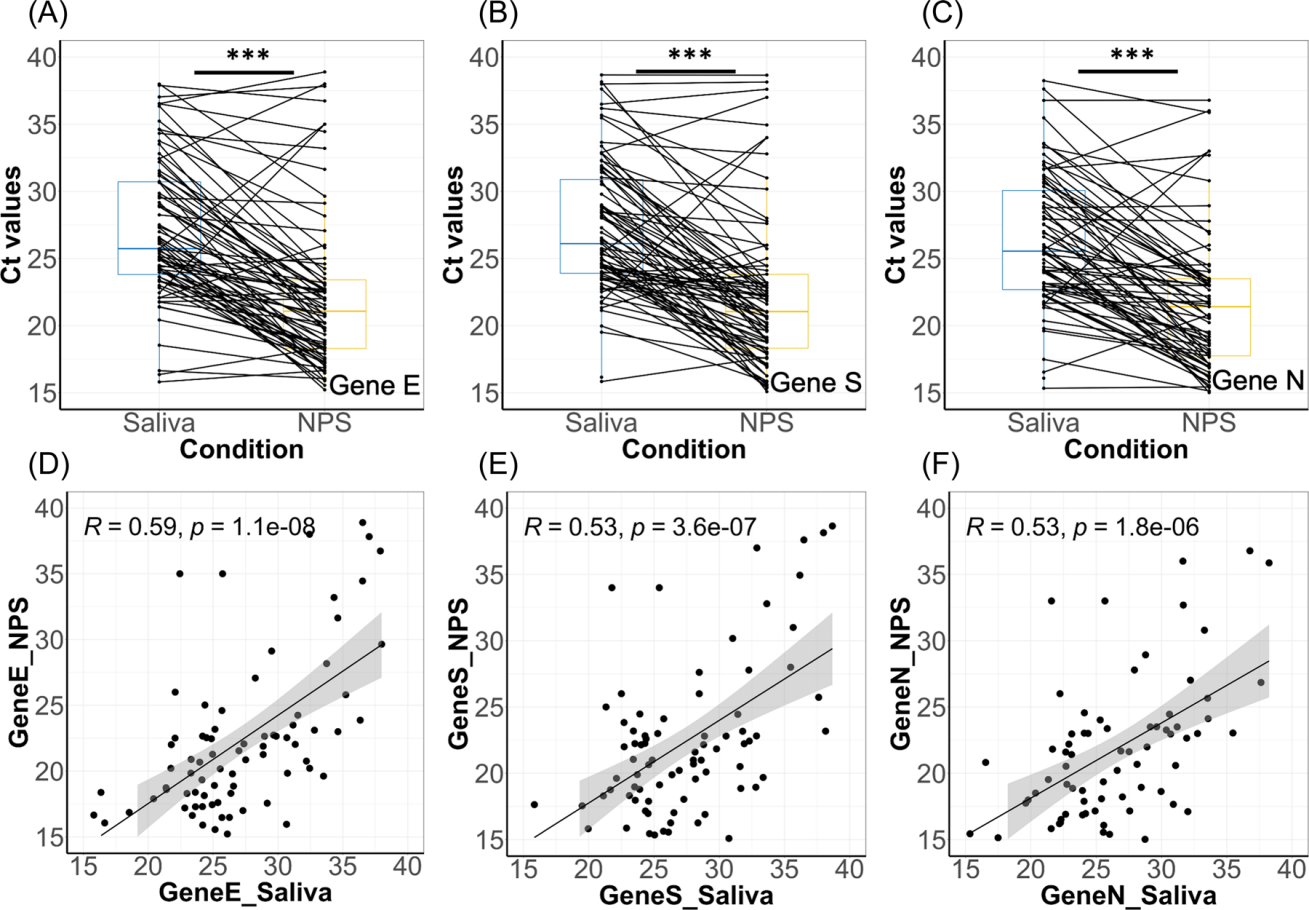
Comparison of Ct values of different genes between the two biofluid samples using the extraction protocol and Allplex™ SARS‐CoV‐2 kit. Pairwise analysis of Ct values of Gene E (A), Gene S (B), and Gene N (C) in patient‐matched saliva and NPS samples. Significant differences among tests (Wilcoxon, *p* < 0.0001) are indicated as ***. Correlation of Ct values of Gene E (D), Gene S (E), and Gene N (F) between saliva and NPS samples. NPS, nasopharyngeal swabs; SARS‐CoV‐2, severe acute respiratory syndrome coronavirus 2

We compared 160 (106 COVID+ and 54 COVID−) paired saliva and NPS specimens for SARS‐CoV‐2 PCR test results. Both biofluids were extracted for RNA and then analyzed with the RT‐PCR Allplex™ SARS‐CoV‐2 Assay. The mean cycle threshold (Ct) values of NPS and Saliva a significant ΔCt of + 3.1, + 3.5, + 3.0 for Gene E, RdRp/S and N, respectively (Table 1 and Figure 2A–C; Wilcoxon, *n* = 106, *p* < 0.001). Ct values of each gene between NPS and saliva samples were significantly positively correlated (Figure 2D–F; (Pearson, *n* = 106, *p* < 0.001), suggesting a strong agreement in readout between the two biofluids.

**Table 1:** Comparison of Ct difference of each gene in saliva and NPS samples using RNA extraction protocol and Allplex™ 2019‐nCoV kit

No		NPS			Saliva		
		Ct Gene E	Ct Gene S	Ct Gene N	Ct Gene E	Ct Gene S	Ct Gene N
COVID19+	106	23.9 (13.8–38.9)	23.4 (13.8–39.0)	23.8 (14.0–38.7)	27.1 (15.8–38.0)	27.3 (14.7–38.7)	26.3 (14.9–38.2)
COVID19−	55	–	–	–	–	–	–

Abbreviations: COVID‐19, coronavirus disease 2019; nCoV, novel coronavirus; NPS, nasopharyngeal swabs.

In all three genes, E, RdRp/S, and N, over the whole patient population a mismatch between saliva was visible at Cts higher than 30, indicating either a differential appearance of virus across the two biofluids or a discrepancy due to experimental procedure. Considering the more robust Gene E, the sensitivity of salivary diagnostic as compared to NPS was 83% and vice versa, and the specificity was 94.8% intrinsic to the biofluid. At Ct values of lower or equal 34, there were three and five samples positive for GeneE but not the others in saliva and NPS, respectively. However, when considering samples with Cts lower or equal to 30 (quantitative load identified as noncontagious) the results were the same for all the three genes and the two biofluids were more comparable with matching between saliva and NPS of 100% and a specificity of 100%.

### 3.2 Comparison of SARS‐CoV‐2 detection using directly matched saliva and NPS specimens with heat‐released RNA protocol

**Figure 3:**
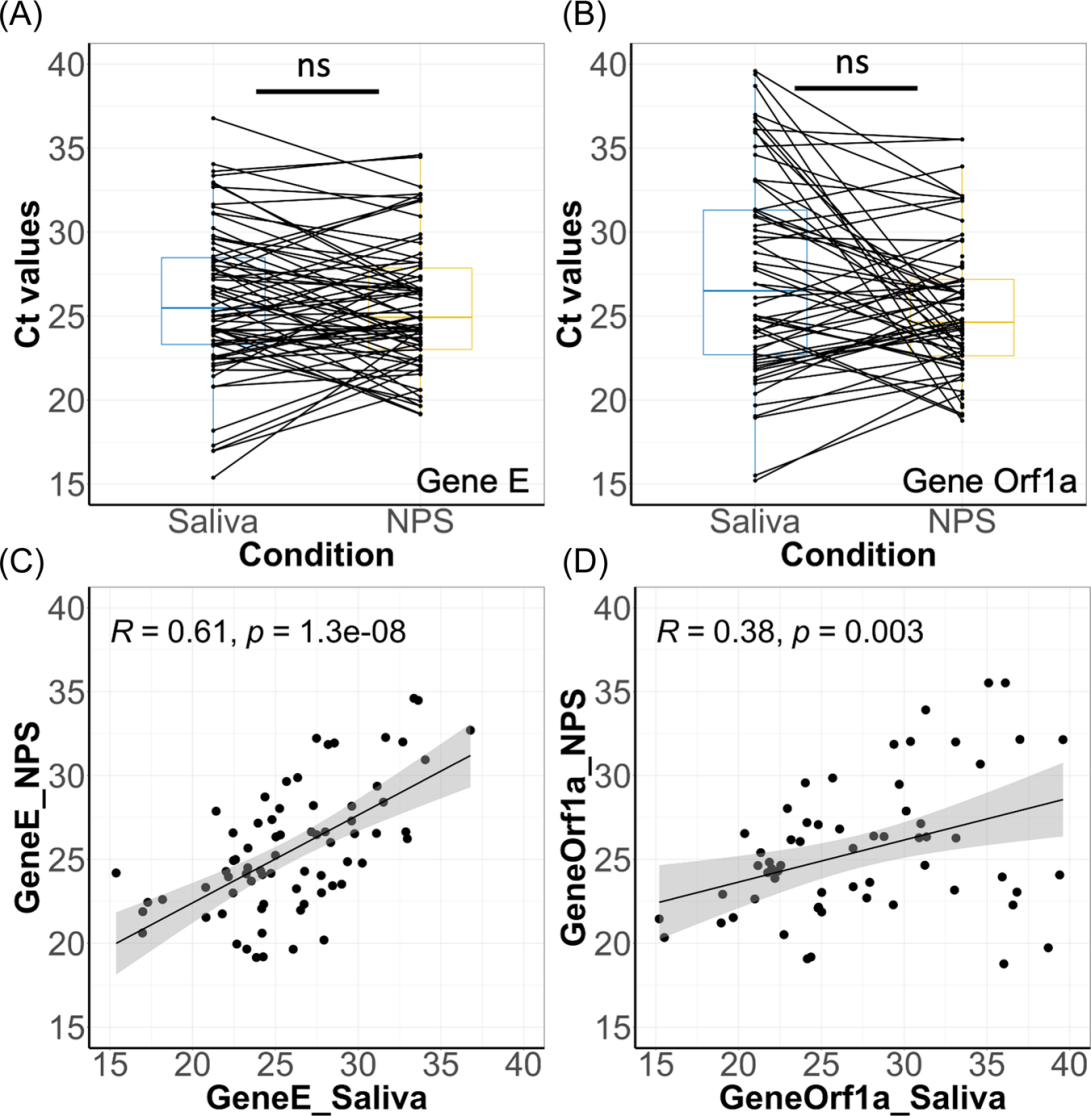
Comparison of Ct values of different genes between the two biofluid samples using the heat‐released RNA protocol and AmpliCube Coronavirus SARS‐CoV‐2 kit. Pairwise analysis of Ct values of Gene E (A) and Gene Orf1a (B) in patient‐matched saliva and NPS samples. Not significant differences among tests (Wilcoxon) are indicated as ns. Correlation of Ct values of Gene E (C) and Gene Orf1a (B) between saliva and NPS samples. NPS, nasopharyngeal swabs; SARS‐CoV‐2, severe acute respiratory syndrome coronavirus 2

Seventy‐six COVID+ and 40 COVID− NPS were matched to Saliva using an adapted version of the heating method^13^ and the AmpliCube Coronavirus SARS‐CoV‐2 kit. While there is no difference in the range of Ct values for the Gene E and Orf1ab between these two biofluids (Table 2 and Figure 3A,B) suggesting that viral titers are comparable between biofluids, and differences depend on the type of RNA‐extraction. A significant positive correlation between Ct values of Gene E (Pearson, *n* = 76, *p* < 0.001) was found between the two biofluids samples but not with Gene Orf1a (Figure 2C,D).

**Table 2:** Comparison of Ct difference of each gene in saliva and NPS samples using heat‐released RNA protocol and AmpliCube coronavirus SARS‐CoV‐2 kit

Number		NPS		Saliva	
		Ct Gene E	Ct Gene Orf1a	Ct Gene E	Ct Gene Orf1a
Covid_19+	76	25.8 (19.2–34.6)	25.5 (18.8–35.5)	25.8 (13.5–36.8)	27.7 (13.7–41.7)
Covid_19−	40	–	–	–	–

Abbreviations: COVID‐19, coronavirus disease 2019; NPS, nasopharyngeal swabs; SARS‐CoV‐2, severe acute respiratory syndrome coronavirus 2.

The comparison between COVID + NPS and saliva samples show only two mismatches between Saliva and NPS considering both genes E and Orf1ab, reporting a 97.2% match over the whole range of Ct values. Considering the more robust Gene E, the sensitivity of salivary diagnostic as compared to NPS using the whole range of Ct values is 98.7% and a specificity of 100%.

### 3.3 Comparison of results obtained with Allplex SARS‐CoV‐2 kit using RNA extraction protocol vs heat‐released RNA protocol with AmpliCube coronavirus SARS‐CoV‐2 kit

**Figure 4:**
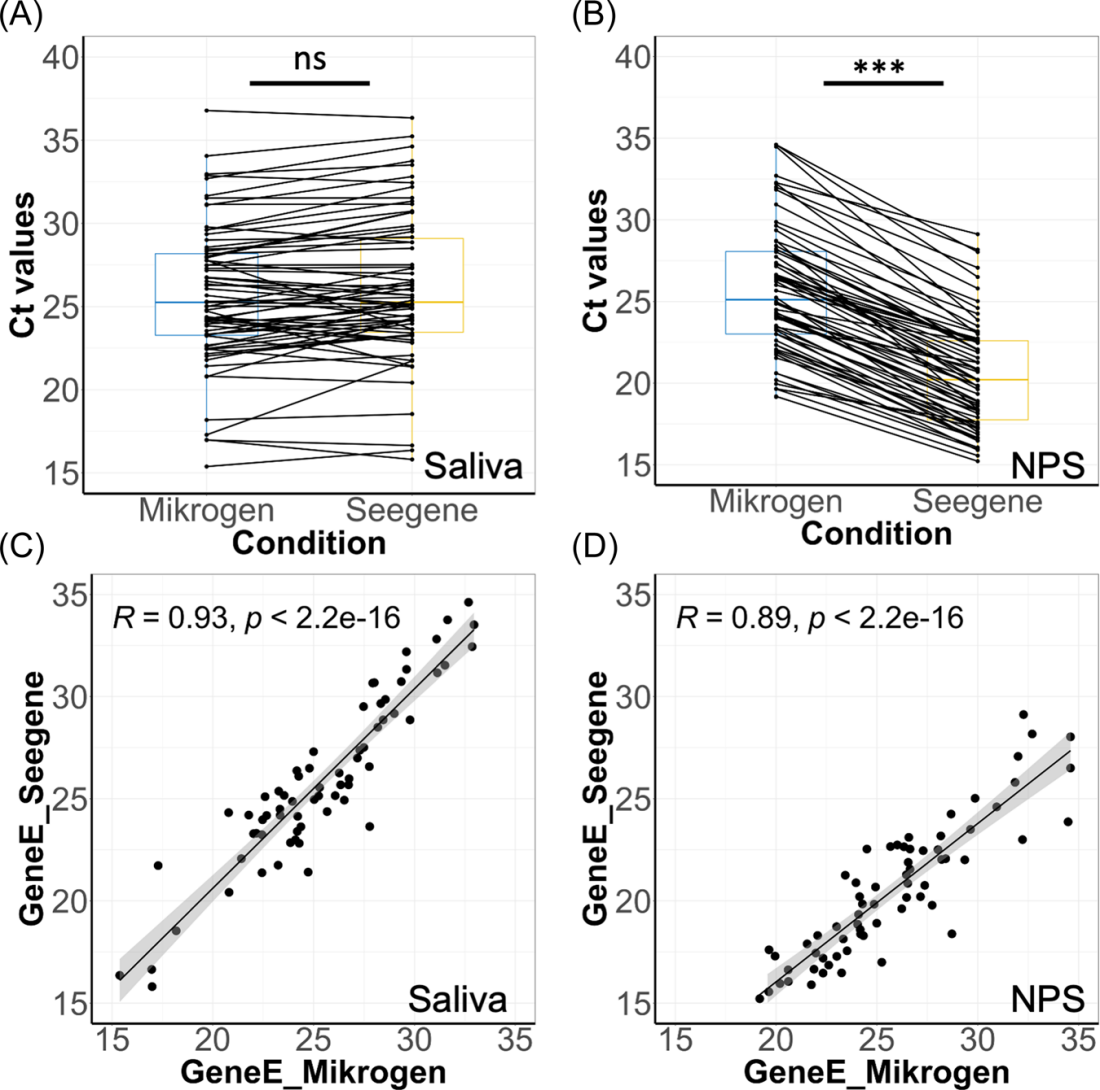
Comparison of Ct values of Gene E using the magnetic beads extraction (Seegene kit) or heat‐release method (Mikrogen kit) on both biofluids. Pairwise analysis of Ct values of Gene E between two different pre‐analytical methods in patient‐matched saliva (A) and NPS samples (B). Not significant differences among tests (Wilcoxon) are indicated as ns, whereas significant differences among tests (Wilcoxon, *p* < 0.0001) are indicated as ***. Correlation of Ct values of Gene E (C) and Gene Orf1a (B) between saliva and NPS samples. NPS, nasopharyngeal swabs

We compared two different methods for extracting RNA: heat‐released RNA and conventional RNA extraction with respect to hands‐on‐time and speed. We used 129 saliva samples and 131 NPS and treated them by RNA‐extraction using magnetic beads or heating to liberate RNA from crude saliva or NPS. RT‐PCR was performed using the Allplex SARS‐CoV‐2 kit and AmpliCube Coronavirus SARS‐CoV‐2 kit for extracted RNA and heated RNA,^13^ respectively. The two preanalytical preparations indicate that the heat‐released RNA method provides a better viral yield returning more positive cases both in saliva (74 vs. 71) and NPS (76 vs. 73) (Table 3). Considering the common Gene E detected by the two kits, heating of NPS showed a significant ΔCt of −5 intrinsic to the concentration of the virus with RNA extraction (Table 3 and Figure 4B), which is not the case for saliva (Table 3 and Figure 4A) and suggests that magnetic beads‐RNA extraction concentrate better NPS than saliva. Correlation analysis of sample pairs that both tested positive confirmed that both extraction and heating methods are in good agreement (Figure 4C,D).

**Table 3:** Comparison of Ct difference of each gene in saliva and NPS samples using RNA extraction with Seegene kit versus heating

		Extracted				Heated		
		*N*	(Ct GeneE)	(Ct GeneS)	(Ct GeneN)	*N*	(Ct Gene E)	(Ct Gene Orf1ab)
Saliva	COVID‐19+	71	26.1 (15.8–36.3)	26.4 (14.7–38.2)	25.5 (14.9–37.6)	74	25.8 (13.5–36.8)	27.7 (13.7–41.7)
	COVID‐19−	58	–	–	–	55	–	–
NPS	COVID‐19+	73	20.4 (13.8–29.1)	20.5 (14.0–30.2)	20.0 (13.8–28.9)	76	25.8 (19.2–34.6)	25.5 (18.8–35.5)
	COVID‐19−	58	–	–	–	55	–	–

Abbreviations: COVID‐19, coronavirus disease 2019; NPS, nasopharyngeal swabs.

### 3.4 Comparison of heat‐released RNA protocol vs RNA column‐extraction protocol for saliva, NPS, and mouthwash

**Figure 5:**
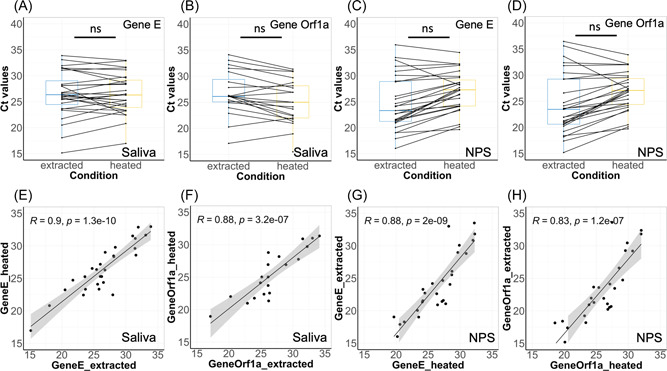
Comparison of Ct values of Gene E and Orf1ab, using the column‐extracted or heat‐release method on both biofluids. Pairwise analysis of Ct values of Gene E and Gene Orf1a between two different pre‐analytical methods in patient‐matched saliva (A and B) and NPS samples (C and D). Not significant differences among tests (Wilcoxon) are indicated as ns. Correlation of Ct values of Gene E and Gene Orf1a between saliva (E and F) and NPS samples (G and H). NPS, nasopharyngeal swabs

We used 30 COVID + saliva, 30 COVID + NPS and 130 mouthwash samples. The specimens were either extracted for RNA using a filter spin column system or heated to liberate RNA from crude saliva. RT‐PCR was performed using the AmpliCube Coronavirus SARS‐CoV‐2 kit. The two preanalytical preparations indicate that heating provides a better viral yield returning more positive cases both in saliva (30 vs. 28) and NPS (30 vs. 28) (Table 4) and both procedure in the two biofluid yield comparable results as expressed in Ct values of the Gene E and Orf1ab (Figure 5A–D). Correlation analysis of sample pairs that both tested positive confirmed that both extraction and heating methods are in good agreement (Figure 5E–H).

**Table 4:** Comparison of Ct difference of each gene between extracted RNA and heated RNA in saliva, NPS samples, and mouthwash samples

		Extracted RNA			Heated RNA		
		N_o_	Ct Gene E	Ct Gene Orf1a	N_o_	Ct Gene E	Ct Gene Orf1a
Saliva	Covid19+	28	26.5 (15.2 ‐ 33.9)	26.2 (14.4 ‐ 34.1)	30	27 (17.0 ‐ 34.1)	25.9 (15.5 ‐ 34.6)
	Covid19−	14	–	–	12	–	–
NPS	Covid19+	28	24.4 (16.0 ‐ 36.0)	24.3 (15.2 ‐ 36.5)	30	27.2 (19.7 ‐ 34.6)	27.0 (18.5 ‐ 35.5)
	Covid19−	15	–	–	13	–	–
Mouthwash	Covid19+	70	32.1 (25.6 ‐ 37.8)	33.0 (26.0 ‐ 38.5)	47	33.5 (27.7 ‐ 37.3)	33.9(25.6‐ 37.8)
	Covid19−	60	–	–	83	–	–

Abbreviations: COVID‐19, coronavirus disease 2019; NPS, nasopharyngeal swabs.

On the other hand, using the mouthwash as a sample type in the RNA heat‐released method failed to return satisfactory results. Comparison of the heat‐released RNA method to RNA extraction method yielded only a 67.1% agreement. Twenty‐three over 70 positive samples were missed using the former method with mouthwash specimens (Table 4).

Overall, the better performance of the extraction‐free pre‐analytical method for RT‐PCR applies only to crude saliva and not mouthwash, prompting us to use in native saliva only for salivary detection of SARS‐CoV‐2 in clinical settings for mass‐screening.

### 3.5 Pooling saliva samples for mass population screening

**Figure 6:**
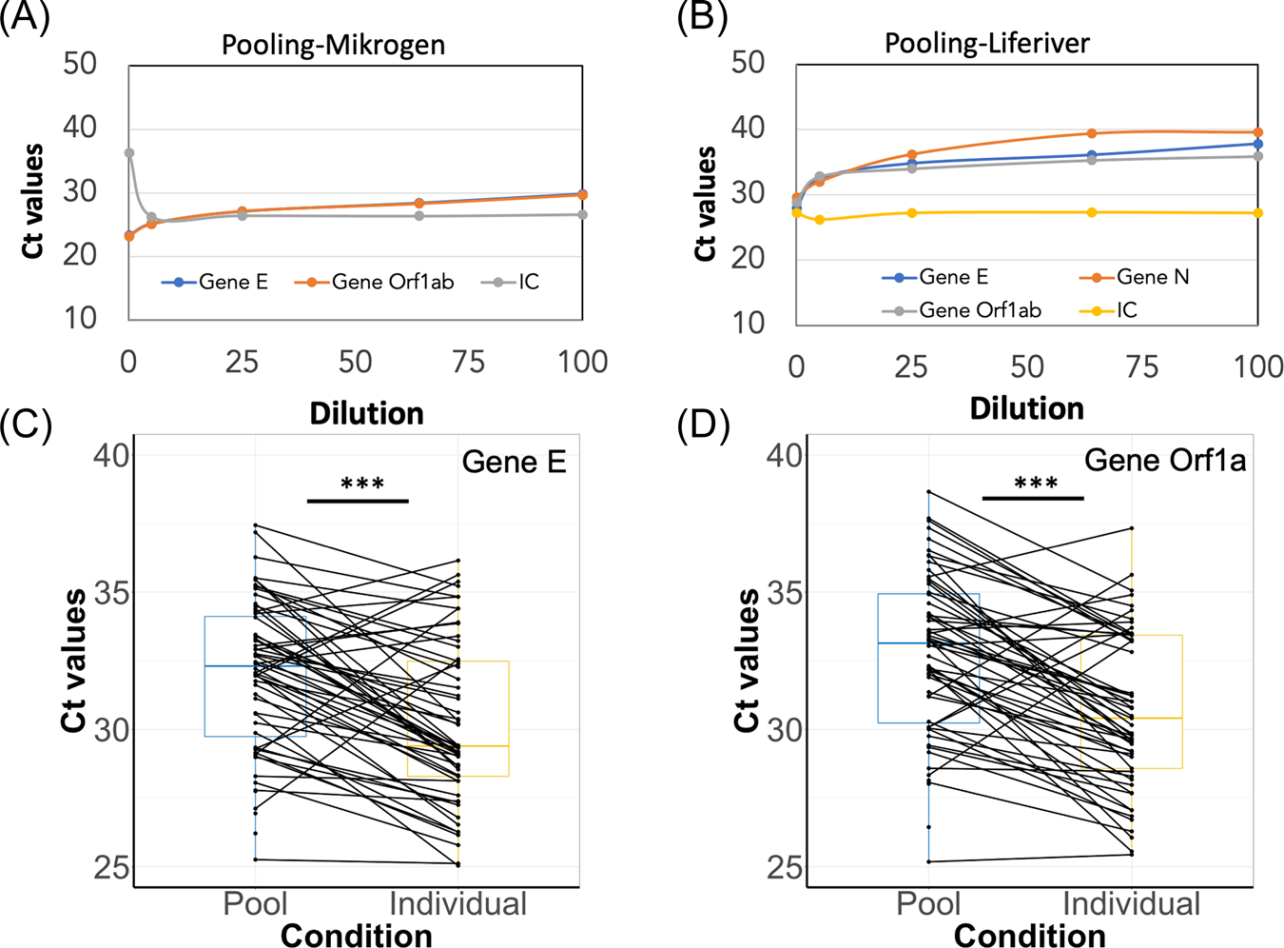
Dot Plot showing the Cts distribution for SARS‐Cov‐2 genes based on pools of 5, 25, 60, and 100 using the AmpliCube Coronavirus SARS‐CoV‐2 kit (Gene E and Gene Orf1ab) (A) and Liferiver RT‐PCR kit (Gene E and gene N and Orf1ab) (B) with IC as constant for both kits. Pairwise analysis of Ct values of Gene E (C) and Gene Orf1a (D) in COVID‐19 detectable pools of eight and individual samples. Significant differences among tests (Wilcoxon, *p* < 0.0001) are indicated as ***. RT‐PCR, reverse‐transcription polymerase chain reaction; SARS‐CoV‐2, severe acute respiratory syndrome coronavirus 2

To confirm the reliability of the bulk pooling method, two RT‐PCR kits from Migrogen and Liferiver were used. The experiment was performed three times using specimens with Ct values between 26 and 27 for Gene E, Gene N, and Orf1ab. The dilution used are respectively: 01 COVID+, five times diluted (1 COVID+ and 4 COVID−), 25 times diluted (1 COVID+ and 24 COVID−), 64 times diluted (1 COVID+ and 63 COVID−) and 100 times diluted (1 COVID+ and 99 COVID−). The results showed that the dilution increases the Ct values for genes E and Orf and genes E, N, and Orf1ab in both Mikrogen (Table 5, Figure 6A) and Liferiver (Table 5, Figure 6B) kits, respectively, in an asymptotic way as expected by the RT‐cycling method. The results confirmed the reliability of using pooling systems 5 (Delta of 1.9 and 3.6 Cts compared with individual by using Mikrogen and Liferiver kits, respectively) to 25 (Delta of 3.9 and 6.2 Cts compared to individual by using Mikrogen and Liferiver kits, respectively) to perform mass screening for example in schools (Table 5, Figure 6). Once a positive pool is identified, all samples have to be tested individually. This is only possible when the pooling is performed in the laboratory and not externally.

**Table 5:** Ct values of each gene in different pooling strategies by using two different commercial RT‐PCR kits from Mikrogen and Liferiver

Dilution	Mikrogen kit			Liferiver kit			
	Ct GeneE	Ct GeneOrf1a	Ct IC	Ct GeneE	Ct GeneN	Ct GeneOrf1a	Ct IC
0	23.3	23.2	36.3	28.0	29.6	28.8	27.2
5	25.2	25.1	26.3	32.5	32.0	32.8	26.2
25	27.1	27.1	26.4	34.8	36.2	34.0	27.2
64	28.4	28.3	26.4	36.1	39.4	35.3	27.3
100	29.8	29.7	26.6	37.8	39.6	35.9	27.2

Abbreviation: RT‐PCR, reverse‐transcription polymerase chain reaction.

We then applied the saliva pooling strategy in school mass testing in Fribourg, Switzerland in 2022. The Ct values of Gene E and Gene Orf1a of 60 pools of 8 containing 1 positive specimen were compared to that of individual specimens (Figure 6C,D). The average Ct values of Gene E and Gene Orf1a in 60 pools of 8 were 32.0 (range: 25.3–37.3) and 32.7 (range: 25.2–38.7), respectively, which were about 2.1–2.4 Cts higher than those in individual specimens. These differences were significant (Figure 6C,D; Wilcoxon, *p* < 0.05). Our analysis indicates that among a sampling of 100 pools of eight samples using the Mikrogen kit a 98% sensitivity and 100% specificity are attained.

## 4 DISCUSSION

The COVID‐19 pandemic has raised an urgent need of searching for fast and reliable scientific procedures, rapid result delivery and proper sample collection. In this study, we considered the important advantages of using saliva specimens in SARS‐CoV‐2 detection. The saliva has our attention due to its several advanced characteristics: (1) noninvasive specimen, (2) easier and safer collection, (3) a good reservoir of viruses, (4) possibility for self‐collection at home, and (5) its convenience for repetitive collection in both adults and children. Saliva has previously proved to be an ideal organic fluid for isolation of proteins, peptides, and viral shedding via many molecular assays.^15^ Many other publications have positive support with scientific proof for using saliva in SARS‐CoV‐2 detection.^6^, ^7^, ^16^, ^17^, ^18^

By applying head‐to‐head comparisons between NPS and saliva using different protocols, we demonstrated an excellent agreement of saliva to NPS, suggesting saliva is an important alternate biological matrix for SARS‐CoV‐2 detection by RT‐PCR. In this study, we found a high concordance between NPS and saliva testing, accounting for 96.2%–97.2% with two different pre‐analytical protocols: bead‐based RNA extraction and heat‐released RNA, respectively. The same results of high concordance range from 87%–100% were found in other studies.^6^, ^18^ However, there are other papers observing the discordance between saliva and NPS specimens. The explanation for these discordances could be due to the different concentration of viral load in these two biofluids at the different stages of infection that samples were collected.^19^ In fact, in this study, we examined different Ct values between saliva and NPS samples with a higher Ct value (lower viral load) in saliva samples compared to NPS when using RNA extraction as the pre‐analytical protocol. As we do not observe any difference using heat‐released RNA method between NPS and saliva we conclude that bead‐based RNA extraction is better suited for concentrating RNA in NPS than saliva while the heating method has comparable applicability in both biofluids, increasing reproducibility of the results.

Several biases could be addressed in using saliva samples from collection to analysis. While our sampling was not time‐locked nor controlled, the possibility remains that toothpaste traces, or other oral chemicals residues in saliva might interfere with the results when using the direct heating RT‐PCR method. In the laboratory, when pipetting saliva samples, the viscosity limits the use of automatized systems for dispensing. The use of EDTA in the TBE solution used in the heating procedure can interfere with the RT‐polymerase activity. In addition, as shown saliva COVID‐19 molecular diagnosis is sensitive to the extraction method, with spin columns as less suitable than the bead‐based or heating‐based RNA extraction process.

In this study we also found a heating method compared to the extraction method, the heat treatment assay allows (1) faster testing time within a very short time (3–3.30 h vs. 5.30–6 h), (2) cheaper when no chemical extraction kits are used, (3) application in low‐resource countries. The consequences of this are that it could reduce testing costs, time cost and increase the efficiency of analytical platforms during the period of mass testing.

To sum up, our findings show that a simple heat shock SARS‐CoV‐2 RT‐qPCR diagnosis method without RNA extraction is a reliable alternative to detect potentially infectious SARS‐CoV‐2‐positive patients at the time of testing. This affordable protocol can help overcome the cost and supply shortages for first‐line SARS‐CoV‐2 diagnosis, especially in developing countries. Our adapted preanalytical heating procedure improves the detection of SARS‐Cov‐2 in saliva as compared to the original study, with a 100% match and Ct values only 1 value apart as compared to RNA extracted material. Using RT‐PCR on individual samples we obtain a robust sensitivity above 98.5% for salivary COVID testing and 100% specificity using two commercially available kits.

The direct method has also the advantage of allowing pre‐analytical pooling with high reliability using groups of 5‐25 when doing mass surveillance in the asymptomatic population, taking a prevalence of the virus below 1%. Our experience indicates that pools of 8 saliva specimens provide an optimal balance between costs and sensitivity for mass‐testing of SARS‐Cov‐2 in asymptomatic children. Another study previously showed similar results with saliva pools of 5 and 10 suitable with a disease prevalence of 9%.^20^

Overall, our study shows that saliva is a reliable biofluid for COVID‐19 detection. Saliva coupled with the direct heating method can also be used as an efficient tool for mass testing, since hundreds of samples can be diagnosed in a few hours, obtaining reliable results. This mass testing should be applied for repetitive testing in hospitals, schools, companies, and public organizations with great convenience for the subjects and at a limited cost when using pooled samples.

## 5 CONCLUSIONS

In our study, we presented the accuracy of salivary COVID diagnosis compared to the conventional NPS, the so‐called golden standard for COVID testing by performing RT‐PCR. Our study highlights that saliva coupled with the heat‐released RNA method represents a very reliable tool in routine COVID‐19 diagnostics. The results of the study indicate that direct saliva molecular diagnosis provides a 99% diagnostic accuracy in detecting SARS‐Cov‐2 infections and is a suitable replacement for NPS RT‐PCR. A pooling method on saliva samples can also effectively apply in mass testing strategy as monitoring SARS‐CoV‐2 will remain a public health need in the coming years.

COVID‐19 salivary diagnostics sets the stage for future molecular diagnosis of pathogens which harbor the oral cavity and are shared with the respiratory tract, based on anatomical proximity.

## AUTHOR CONTRIBUTION STATEMENT

**Hanh Nguyen‐Kim**: Data analysis and interpretation; writing original draft; review and editing. **Christiane Beckmann**, **Maurice Redondo**, **Jérémy Ziliox**, and **Virginie Vallet**: Sample analyses in VIOLLIER. **Karin Berger‐Sturm** and **Jan Von Overbeck**: Recruitment of participants; sample collection. **Lavinia Alberi Auber**: Conceptualization; review and editing. All authors approved the final version of the manuscript for publication.

## CONFLICTS OF INTEREST

The authors declare no conflicts of interest.

## Data Availability

Data are available on request from the authors. The data that support the findings of this study are available from the corresponding author upon reasonable request.
